# Supplementary material for: CRISPR/Cas-9 mediated knock-in by homology dependent repair in the West Nile Virus vector Culex quinquefasciatus Say
Source: Sci Rep. 2021 Jul 22;11:14964. doi: 10.1038/s41598-021-94065-z (PMC8298393; doi:10.1038/s41598-021-94065-z)
Supplement: Supplementary file 1 — Supplementary Information. [file 41598_2021_94065_MOESM1_ESM.docx]

**Additional Information**


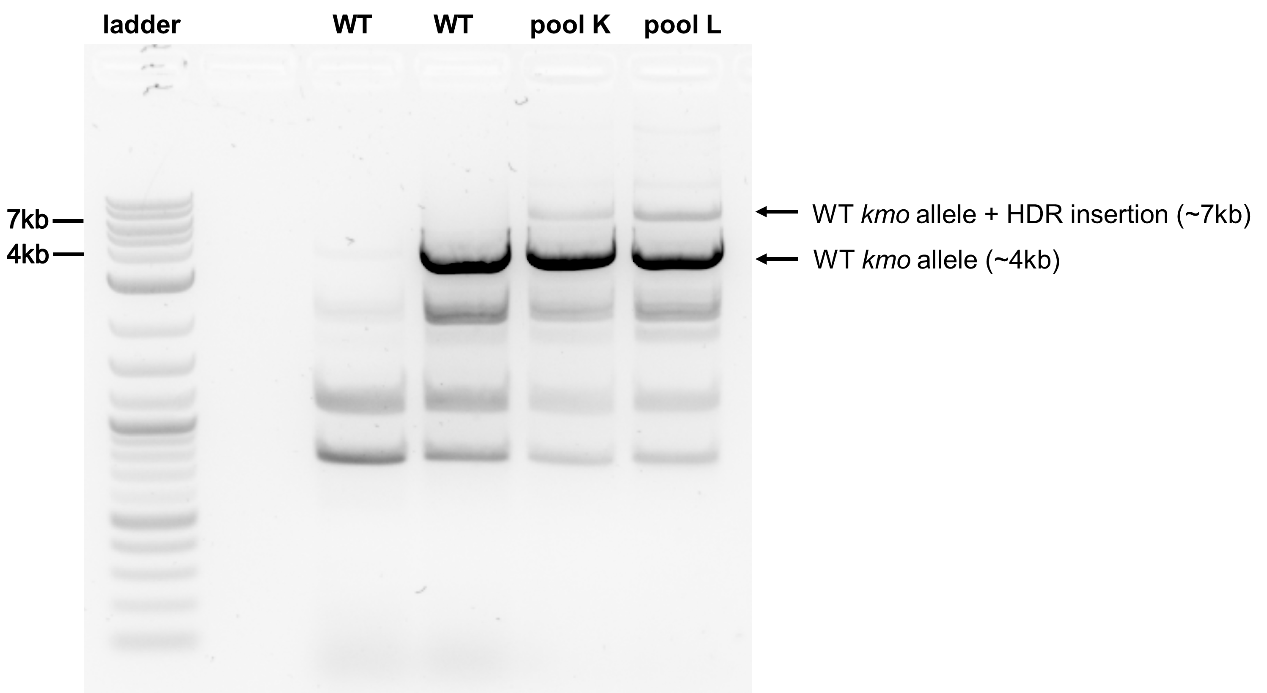


**Figure S1. PCR confirmation of the *kmo* knock-in.** Successful integration of the HDR construct into the *kmo* locus produced expected banding patterns along with additional seemingly non-specific amplicons. The amplicons of expected size for the *kmo* alleles were excised and sequence confirmed.

**
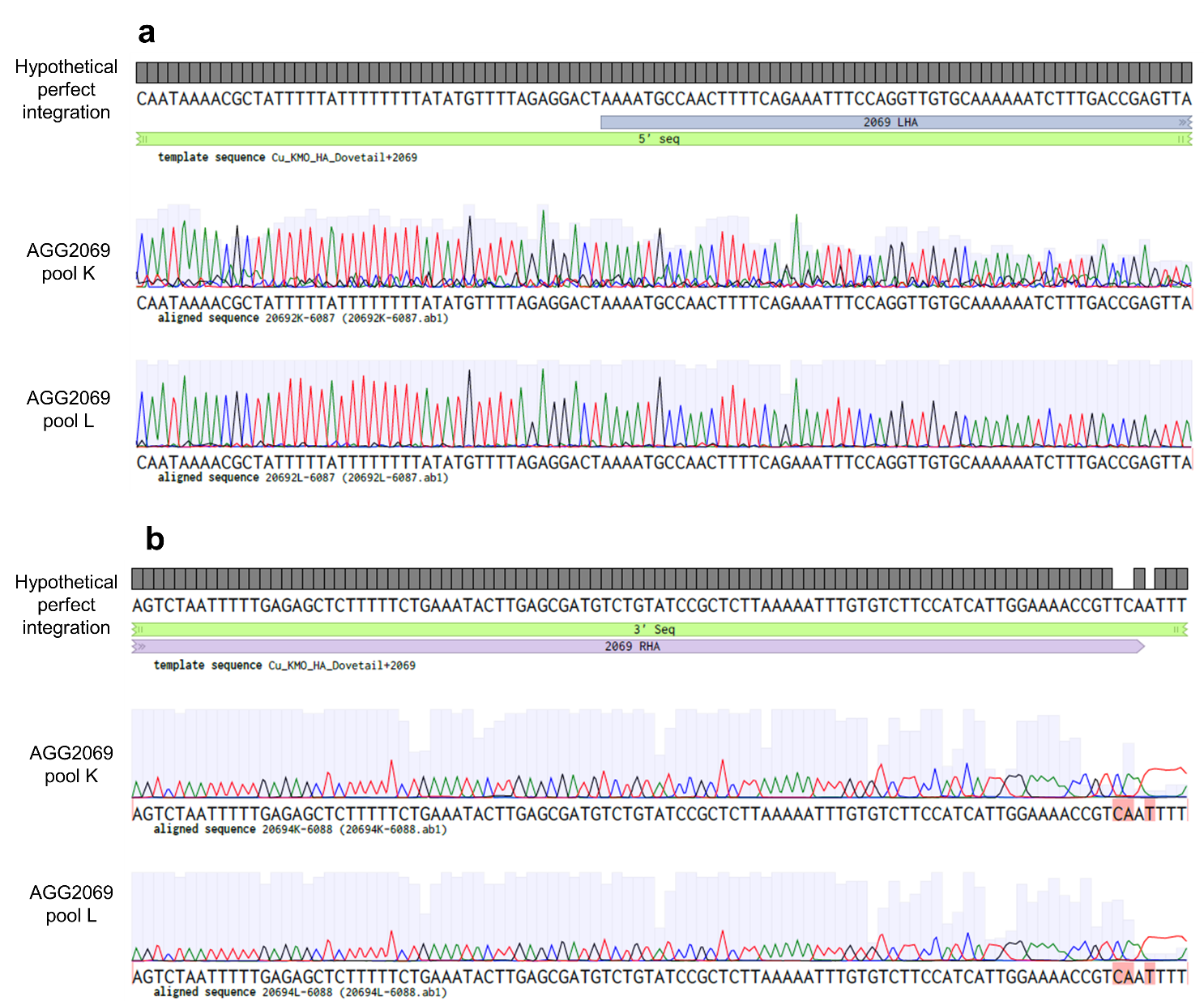
**

**Figure S2. Sequence confirmation of the *kmo* knock-in.** Sanger sequencing traces showing (a) the 5’ and (b) the 3’ junctions of the *kmo* knock-in cassette within the genome. In the 3’ junction, the reverse primer binds to the genomic region immediately adjacent to the right homology arm which resulted in poor quality basecalling (red highlighted bases) at the junction of the homology arm with the genome (b).

**
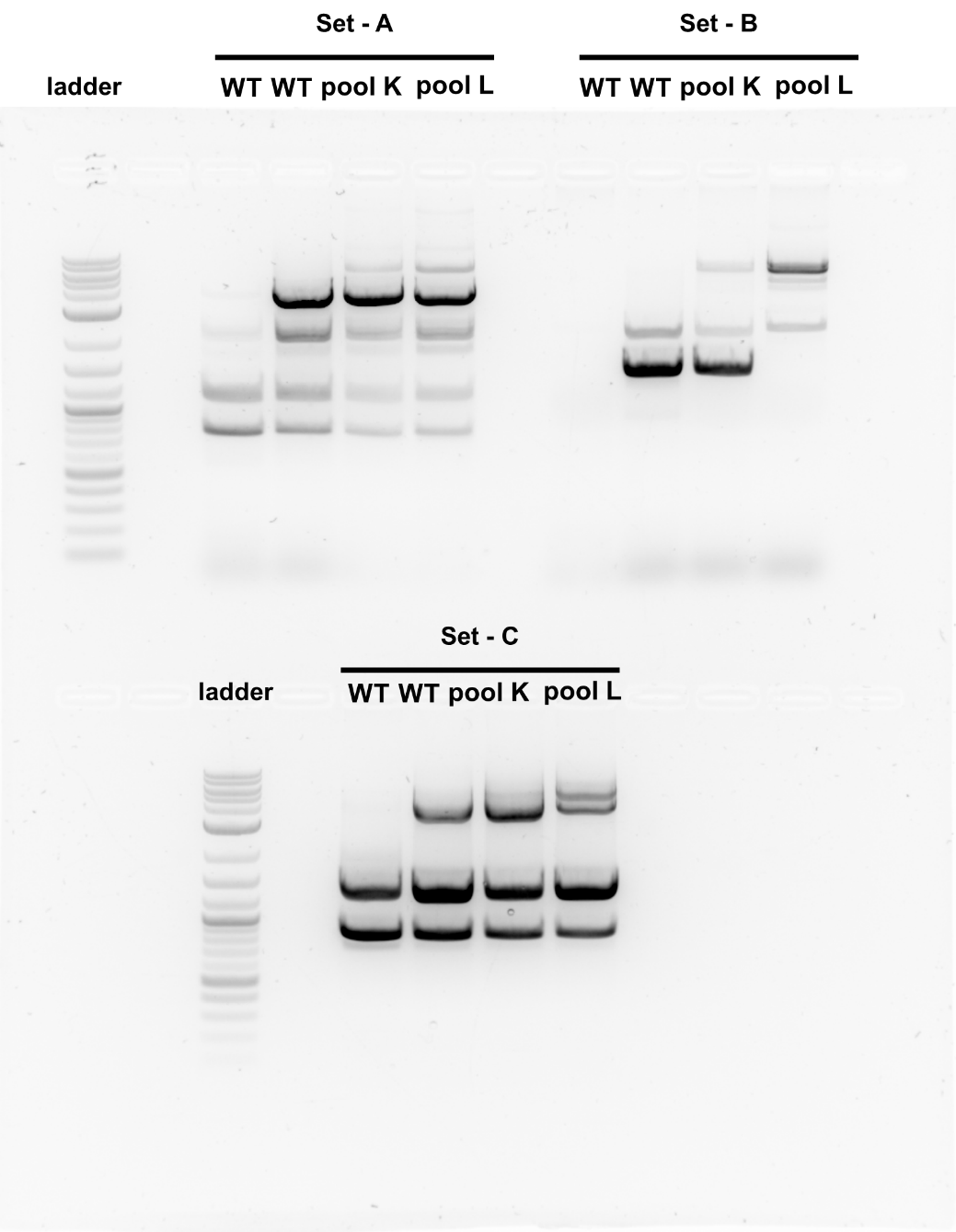
**

**Figure S3. Testing of wild type *kmo* PCR primers.** Set A, B & C – three different pairs of primers. PCR Primer Set-A gave the amplicons of expected size and the cropped image shown in supplementary figure S1.

**
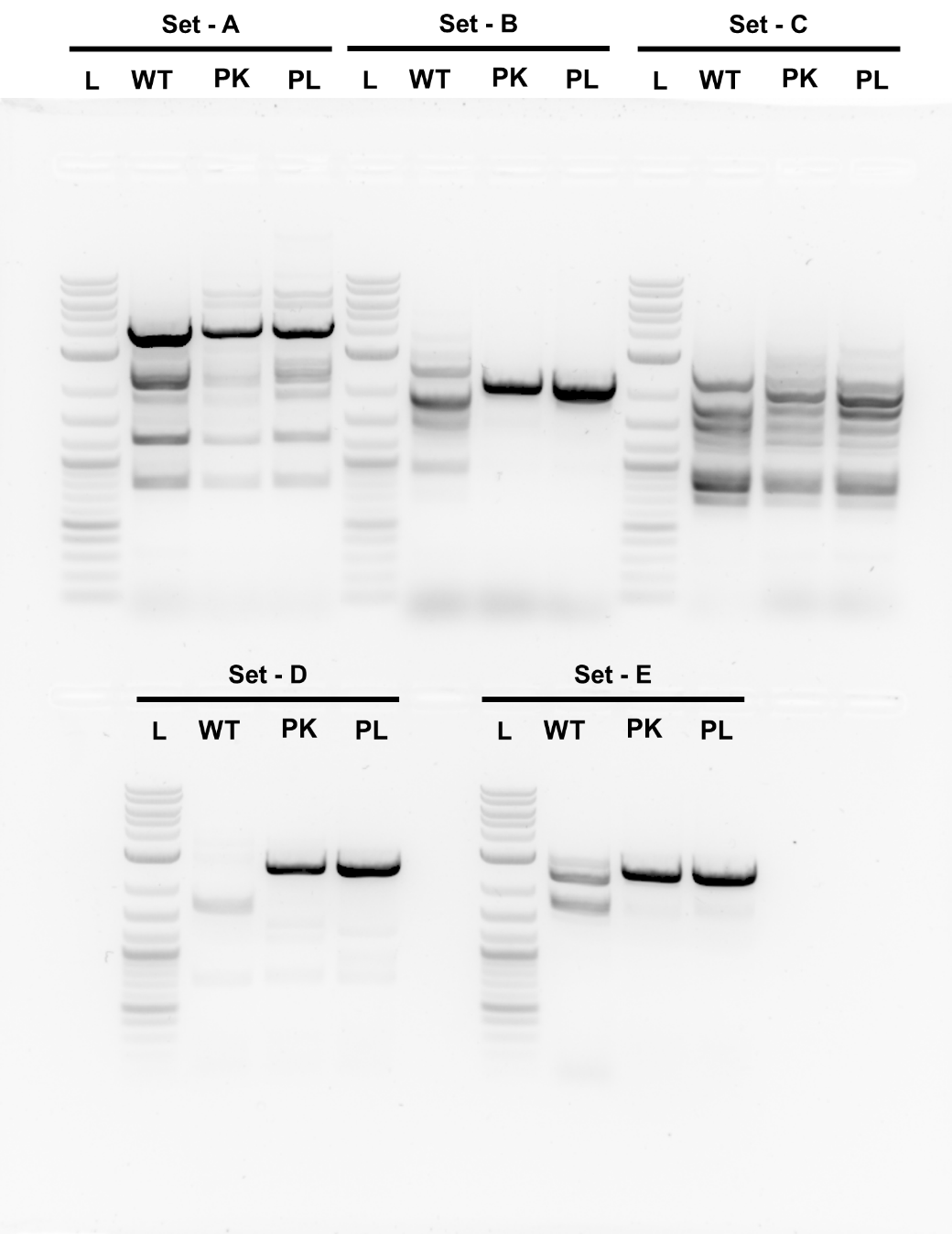
Figure S4. Testing of PCR primers of *kmo knock-in.*** Set A, B & C – three different pairs of primers for amplification of Left Homology arm. Set – D & E – PCR primer pairs for amplifying RHA arm. PCR primer Set – B & D gave the amplicons of expected size and the cropped image shown in the figure 2.

**Competing interests**

The authors declare no competing interests.
